# A Co-Designed, Culturally-Tailored mHealth Tool to Support Healthy Lifestyles in Māori and Pasifika Communities in New Zealand: Protocol for a Cluster Randomized Controlled Trial

**DOI:** 10.2196/10789

**Published:** 2018-08-22

**Authors:** Marjolein Verbiest, Suaree Borrell, Sally Dalhousie, Ridvan Tupa'i-Firestone, Tevita Funaki, Deborah Goodwin, Jacqueline Grey, Akarere Henry, Emily Hughes, Gayle Humphrey, Yannan Jiang, Andrew Jull, Crystal Pekepo, Jodie Schumacher, Lisa Te Morenga, Megan Tunks, Mereaumate Vano, Robyn Whittaker, Cliona Ni Mhurchu

**Affiliations:** ^1^ National Institute for Health Innovation School of Population Health University of Auckland Auckland New Zealand; ^2^ Toi Tangata Auckland New Zealand; ^3^ The Fono Auckland New Zealand; ^4^ Centre for Public Health Research Massey University Wellington New Zealand; ^5^ South Waikato Pacific Islands Community Services Trust Tokoroa New Zealand; ^6^ School of Nursing University of Auckland Auckland New Zealand; ^7^ Department of Human Nutrition University of Otago Dunedin New Zealand

**Keywords:** behavior change, randomized controlled trial, co-design, mHealth, health behavior, noncommunicable diseases, New Zealand, Māori, Pasifika

## Abstract

**Background:**

New Zealand urgently requires scalable, effective, behavior change programs to support healthy lifestyles that are tailored to the needs and lived contexts of Māori and Pasifika communities.

**Objective:**

The primary objective of this study is to determine the effects of a co-designed, culturally tailored, lifestyle support mHealth tool (the OL@-OR@ mobile phone app and website) on key risk factors and behaviors associated with an increased risk of noncommunicable disease (diet, physical activity, smoking, and alcohol consumption) compared with a control condition.

**Methods:**

A 12-week, community-based, two-arm, cluster-randomized controlled trial will be conducted across New Zealand from January to December 2018. Participants (target N=1280; 64 clusters: 32 Māori, 32 Pasifika; 32 clusters per arm; 20 participants per cluster) will be individuals aged ≥18 years who identify with either Māori or Pasifika ethnicity, live in New Zealand, are interested in improving their health and wellbeing or making lifestyle changes, and have regular access to a mobile phone, tablet, laptop, or computer and to the internet. Clusters will be identified by community coordinators and randomly assigned (1:1 ratio) to either the full OL@-OR@ tool or a control version of the app (data collection only plus a weekly notification), stratified by geographic location (Auckland or Waikato) for Pasifika clusters and by region (rural, urban, or provincial) for Māori clusters. All participants will provide self-reported data at baseline and at 4- and 12-weeks postrandomization. The primary outcome is adherence to healthy lifestyle behaviors measured using a self-reported composite health behavior score at 12 weeks that assesses smoking behavior, fruit and vegetable intake, alcohol intake, and physical activity. Secondary outcomes include self-reported body weight, holistic health and wellbeing status, medication use, and recorded engagement with the OL@-OR@ tool.

**Results:**

Trial recruitment opened in January 2018 and will close in July 2018. Trial findings are expected to be available early in 2019.

**Conclusions:**

Currently, there are no scalable, evidence-based tools to support Māori or Pasifika individuals who want to improve their eating habits, lose weight, or be more active. This wait-list controlled, cluster-randomized trial will assess the effectiveness of a co-designed, culturally tailored mHealth tool in supporting healthy lifestyles.

**Trial Registration:**

Australia New Zealand Clinical Trials Register ACTRN12617001484336; http://www.ANZCTR.org.au/ACTRN12617001484336.aspx (Archived by WebCite at http://www.webcitation.org/71DX9BsJb)

**Registered Report Identifier:**

RR1-10.2196/10789

## Introduction

New Zealand is ranked third in the developed world with respect to obesity rates, with almost one in three New Zealand adults being obese (31.2%) [1]. High body mass index is now the second-ranked avoidable risk to health (after unhealthy diet) for the New Zealand population, accounting for 9.2% of total disability-adjusted life years [2]. Substantial ethnic inequalities exist, with Māori (indigenous New Zealanders; 15% of total population) and Pasifika (collective group of people representing different Pacific Island nations, predominantly from the South Pacific region; 7% of total population) adults living in New Zealand experiencing obesity rates 1.7 and 2.4 times higher than those of non-Māori and non-Pasifika adults, respectively [3].

The determinants of obesity are complex, and Māori and Pasifika are disproportionately affected by socioeconomic and dietary factors that predispose obesity and obesity-related illness [4]. Interventions designed for the general population tend to be less effective for Māori and Pasifika communities [5,6] and may contribute to increased health inequalities [7]. Culturally-tailored interventions are generally more effective [8] and have shown beneficial effects in other countries [9-12]. Nevertheless, funding pressures make it difficult to sustain resource-intensive interventions and maintain long-term health promotion activities.

The broad population penetration of mobile and wireless technologies and advancements in their app offers a potential solution. In total, 92% of New Zealanders own a mobile phone (67% own a mobile phone) [13] and 80% have internet access [14]. Further, there are no significant differences in mobile phone ownership or internet access by ethnicity or education and few differences by age (for those aged <65 years) [13]. Formative research in New Zealand indicates that the majority of Māori would be keen to use an mHealth intervention for weight loss [15], and systematic reviews indicate that mHealth interventions can lead to behavior change [16-19].

mHealth programs (ie, the usage of mobile and wireless technologies designed to achieve medical objectives) [20] have been shown to effectively help people change various health behaviors [19,21-26] and improve other secondary risk factors for cardiovascular diseases, such as blood pressure and medication adherence [27]. Nevertheless, most mHealth programs are designed with minimal input from end-users and lack tailoring to cultural needs. As a result, such interventions often have poor uptake and low rates of use by these communities and end-users [28].

The OL@-OR@ (pronounced “ola ora”) project (ACTRN12617001484336) consists of two stages: a co-design phase and a trial phase. The co-design phase focused on the development of a culturally-tailored, lifestyle support mHealth tool (mobile phone app and website) for Māori and Pasifika communities in New Zealand (conducted between June 2016 and October 2017). Subsequently, a systematic and theory-driven approach was applied to the selection of relevant culturally-tailored behavioral determinants and change techniques, informed by the co-design data gathered. The cluster-randomized trial described in this protocol was co-designed with communities to measure the impact of the OL@-OR@ mHealth tool on key preventable risk factors for noncommunicable disease, specifically diet, physical activity, smoking, and alcohol use.

## Methods

### Co-Design

The trial was designed using the same participatory co-design principles that underpinned the first phase of the OL@-OR@ study. An academic-community partnership has been established guided by the principles of participation and protection and aligned with New Zealand’s Treaty of Waitangi (founding document). Māori- (ie, *kaupapa*) and Pasifika-specific research approaches have been applied throughout the design of the mHealth tool and the trial [29]. Toi Tangata, a Māori health promotion provider, led the engagement process with Māori (involving communities in the Wellington and Auckland regions), and two Pasifika health providers, The Fono in Auckland and South Waikato Pacific Islands Community Services Trust in Tokoroa, led the engagement processes with their local communities. This paper focuses on the OL@-OR@ trial phase. During several *hui* (meetings) attended by Māori and Pasifika community representatives and academics, decisions were made regarding the trial design, including the community-based cluster trial design, the control condition, primary and secondary outcome measures, recruitment methods, and timelines.

### Study Design

This trial protocol adheres to the SPIRIT guidelines (Multimedia Appendix 1). A two-arm, parallel, cluster-randomized controlled trial will be conducted in New Zealand between January and December 2018. The aim is to recruit 1280 participants from 64 clusters (32 Māori, 32 Pasifika); 32 clusters per arm (16 Māori, 16 Pasifika); 20 participants per cluster.

### Eligibility Criteria

The clusters consist of Māori or Pasifika groups or communities identified by the community partners (Multimedia Appendix 2). A cluster was defined as any distinct location or setting in New Zealand where likeminded people or those with shared interests congregate, including a church, *marae* (meeting grounds), school, workplace, sports club, or community group. Individual participants are eligible if they are a member of a participating cluster; reside in New Zealand; are aged ≥18 years; have regular access to a mobile phone, tablet, laptop, or computer; have regular access to internet (at least once a week); are able to provide written consent (e-consent); and have an email address or are prepared to create an email account. Although people within clusters may self-identify as both Māori and Pasifika, for the purposes of the analysis, they will be recorded as Māori if they are part of a Māori cluster and as Pasifika if they are part of a Pasifika cluster.

### Recruitment

Recruitment will be community-led, that is, Māori and Pasifika community coordinators will identify eligible clusters and approach a potential leader within these clusters. Once initial contact has been made with the identified cluster leaders, they will be given information on what is required to be involved with the study, including outlining the process of being randomized to control or intervention conditions. Cluster leaders will then provide informed consent for participation of their group or community in the study. The next step will be for the cluster leaders to begin recruiting individual participants for the trial. Specific recruitment methods will include inviting potential participants to face-to-face information sessions on the trial using social media (eg, Facebook), posters, brochures, other advertising material, and word-of-mouth and inviting established networks and groups previously formed with the community partners.

Potential participants will also be able to enroll in the trial if they are invited by an existing trial participant via the OL@-OR@ tool or if they share a phone with a trial participant who has downloaded the OL@-OR@ tool on that phone.

### Study Procedures

People who are identified as part of a cluster by their local community coordinator will be invited to participate in the study. An information meeting will be organized to provide details about the study and answer any questions that potential participants may have. Potential participants will be provided with a copy of the participant information sheet and brochure. Those who are interested and meet the inclusion criteria may sign up for the study with their local community coordinator. Potential participants who indicate interest will be sent an email with study registration details. By clicking on a link in the email, they will be able to complete their registration details and provide e-consent (participants consenting using a computer-based consent form). They will then complete baseline questions online. Once all questions are complete and the terms of use are accepted, participants will be able to download the OL@-OR@ tool (intervention) or a data collection version of the app (control) on their device. If they do not have a mobile phone or tablet, participants will be able to access a Web version through a link provided. All participants will be asked to use the app (or Web version) for 12 weeks and keep the app running on their device for (at least) the whole 12-week period. At 4- and 12-weeks, Web-based follow-up assessments will take place. After 12 weeks, participants in intervention clusters can continue using the app if they wish. Participants assigned to the wait-list control condition will be able to download the full app or use the Web version once they have completed the 12-week questionnaire.

### Randomization, Allocation Concealment, and Blinding

Clusters will be randomly assigned in a 1:1 ratio to either the intervention or control condition using a computer-generated randomization list prepared by the study statistician. Block randomization will be used with variable block sizes of 2 and 4, stratified by locality (Auckland or Waikato) for Pasifika clusters and by region (rural, urban, or provincial) for Māori clusters to ensure these factors are balanced across the two randomized groups. The randomization list will be concealed until the point of randomization for all clusters. Randomization codes will be kept secure in a restricted computer-based project file and will only be accessible to the project manager and project coordinator who will disclose these to community coordinators when clusters have been identified and participants have signed their agreements. Due to the nature of the intervention, it will not be possible to blind participants or research staff to the use of the different intervention conditions of full versus basic (data collection only) version of the OL@-OR@ tool. The risk of contamination between cluster arms is minimized via selection of distinct clusters located in different settings and communities around New Zealand. The OL@-OR@ system has also been designed so that it is not possible for participants in one arm of the trial (control or intervention) to invite participants in the other arm.

### Study Intervention

#### Intervention Design

The OL@-OR@ tool is designed to help Māori and Pasifika and their *whānau* (extended family) to improve their health and wellbeing by making small positive, culturally relevant changes to their lifestyle. Co-design methods were used to capture and understand the needs of members of Māori and Pasifika communities. These methods fostered expression, reflection, and sharing and informed the development of the intervention.

Ethnic-specific models of health and wellbeing [30-33] were used to interpret the co-design data and to select relevant enablers and barriers of health behavior change, behavior change techniques, and intervention features that align with the cultural needs and wants of its users. The Theoretical Domains Framework [34] and Behavioral Change Taxonomy [35,36] were used to map similarities and differences in identified behavioral determinants and change techniques to those confirming that the OL@-OR@ intervention aligns with evidence-based behavior change principles [37].

Feedback from community focus groups on desired content (physical activity, family, and healthy eating) and themes (holistic wellbeing, connecting, motivation and support, and health literacy) were integrated with established behavioral determinants and change techniques to create the mHealth tool content and features. The design features and proposed functions were presented using wireframes. These were taken back to communities in a continuous two-way iterative improvement process until an agreement was reached that the final wireframe design reflected community wants and needs. A cross-platform (iOS, Android, and Web-based) prototype of the app was created in-house by a team of developers using Apache Cordova and Drupal 8 platforms. The final app was released on Google Play and the App Store.

The tool supports users to set goals and specific steps to reach those goals. Users are encouraged to invite others to join them on their journey and can collect online reward tokens as they achieve their goals. The tool also provides information concerning healthy eating, physical activity, local activities, and health services. Lifestyle trackers help users to monitor their progress (see Multimedia Appendix 3 for screenshot examples of tool features). Regular culturally-tailored reminders and motivational messages (4-5 messages each week) are also sent to motivate users to reach their goals. These messages are delivered via notifications through the app and are stored in the message section of the app. At the beginning of each week, one *whakataukī* (motivational message) will be sent. Lifestyle messages including culturally-tailored tips on eating more healthily, doing more physical activity, reducing stress, improving sleep, and weight loss will be sent weekly. Also, any participants who report that they smoke will receive weekly messages about smoking cessation. Participants will also receive messages highlighting how to use specific features of the tool (weekly) and goal reminders at the end of each week reminding them to review or set new goals (see Multimedia Appendix 4 for examples of motivational messages).

#### Control Condition

Clusters assigned to the control condition will receive a basic version of the OL@-OR@ tool. This version will only collect data (baseline and 4-week and 12-week questionnaires) and provide a weekly motivational message thanking participants for partaking in the study and counting down the weeks until they receive the full OL@-OR@ tool at the end of the 12 weeks. The countdown is to help motivate those in the control group to complete the study. At the end of the study, when all assessments are completed, participants in the control clusters will be able to download the full OL@-OR@ tool to use for as long as they wish. Example screenshots from the control app can be seen in Multimedia Appendix 5.

### Recompense for Involvement in the Study

To acknowledge participants’ time and involvement in the study, *koha* (gift or donation) will be available for each cluster (NZ $500 per cluster of 20 participants, prorated per number of participants recruited for clusters of fewer than 20 participants). The community partners will decide the best way to provide *koha* to the communities involved (eg, provision of a voucher, cash for the community, a donation to a named charity, or purchasing of equipment for the community). Some clusters may elect to share the *koha* equally between enrolled participants.

### Baseline Assessments

At baseline, data will be collected from each cluster concerning the community type (eg, a church community or a sports club), predominant ethnicity in the community, approximate number of community members, approximate number of cluster members interested in participating in the study, and cluster leader and contact details.

In addition, the following data will be collected at baseline from each study participant:

- Sociodemographic data: date of birth, gender, ethnicity, hapu (Māori subtribe) and iwi (Māori tribe) where relevant, highest education level, and annual household income.
- Anthropometric data: self-reported weight (in kilograms) and height (in centimeters).
- Health status: self-reported health condition(s) defined as being told by a doctor that they have high blood pressure, high cholesterol, diabetes, or heart disease.
- Physical activity: measured by the Godin Leisure Time Physical Activity Questionnaire [37].
- Smoking behavior: measured by 7-day point prevalence of self-reported smoking abstinence [38].
- Alcohol intake: measured by the Alcohol Use Disorders Identification Test Consumption [39].
- Fruit and vegetable consumption: measured by items used in the New Zealand Health Survey [40].
- Kava consumption: questions include “Do you consume Kava?,” “How often do you consume Kava?,” and “How many Kava drinks do you consume in a typical week?”
- Holistic wellbeing (for Māori participants only): Tūhononga (cultural connections), *Mauri* (life force or essence), wellbeing, whanaungatanga (family wellbeing and social connectedness) and Rangatiratanga (self-determination, motivation, and management), measured by 16 questions informed by Māori health models Te Whare Tapa Whā [30] and Te Pae Mahutonga [27] and adapted in part from the Hua Oranga Māori mental health assessment questionnaire [31]. Answers are measured on a 6-point Likert scale.
- Holistic wellbeing (for Pasifika participants only): spiritual, physical, mental, and family wellbeing measured by 10-items designed for the purpose of this study based on the Fonofale Model [33], the Ottawa Charter and Hua Oranga [32]. All answers are measured on a 5-point Likert scale.
- Pacific and Kiwi-New Zealand Heritage and Lifestyle (Pasifika participants only): Attitudes and beliefs about Pacific and Kiwi-New Zealand heritage and lifestyle measured using an 8-item cultural affiliation questionnaire [41,42]. Answer categories consist of a 5-point Likert scale.

### Primary Outcome Measure

The primary outcome for the trial is participant adherence at 12-weeks to the recommended health guidelines, as defined by a self-reported composite health behavior score based on the European Prospective Investigation into Cancer-Norfolk Prospective Population Study [38] and as used in a previous trial evaluating the effectiveness of an mHealth-delivered comprehensive cardiac rehabilitation program [43]. This composite score includes: smoking (1: not currently smoking, 0: had ≥1 cigarettes in past 7 days), fruit and vegetable intake (1: ≥5 servings daily, 0: ≤4 servings daily), alcohol intake (1: ≤13 units per week, 0: ≥14 units per week), and physical activity (1: ≥14 units of moderate to vigorous activity/week, 0: ≤13 units of moderate to vigorous activity/week). Scores range from 0 to 4 based on the number of health guidelines met. Participants are classified as adherent if they score 3 or more out of 4 and nonadherent if they score 2 or less. These measures are assessed at an individual level but analyzed and reported at a cluster level (as are the secondary measures).

### Secondary Outcome Measures

Secondary outcome measures will be collected at 4- and 12-week follow-up assessments via a Web-based questionnaire and include the same health behavior outcomes as those assessed at baseline (physical activity, smoking, alcohol intake, and fruit and vegetable consumption) and holistic wellbeing. At 12-week follow-up, user engagement and interaction with the mobile phone app will be quantified using an engagement index [44]. The index is an adapted version of the Web Analytics Demystified Visitor Engagement Index [45]. The original index comprised 7 subindices (click depth, loyalty, recency, interaction, feedback, brand, and duration indices). Although measuring all indices is ideal, the Web Analytics Demystified Visitor Engagement Index protocol emphasizes that the calculation can be adapted to suit the project [45]. Therefore, in line with a previous study assessing user engagement of an mHealth intervention [44], we will select relevant Web metrics to develop a composite engagement index for users of the OL@-OR@ tool. These metrics will include session duration, page views per session, and the number of push notifications. They will be used to calculate the following subindices: (1) click depth index: the number of pages participants view per session in the app, (2) loyalty index: the frequency of participants accessing the app after they commence the intervention, (3) interaction index: the number of push notifications sent through the app that are opened, (4) recency index: time lag between each session when the participant accessed the app, and (5) feedback index: self-reported 20-item measure of participant satisfaction with the app (questions relate to ease of navigation, usefulness of information, helpfulness of notifications, and satisfaction with the look of the app) [44].

The overall engagement index summarizes the subindices from date of registration to 12-week follow-up. The overall index will provide a score for each participant that measures overall engagement with the app during this period. Cutoff points will be developed based on the distribution of the total sample’s index scores using tertiles. Participants will then be categorized as either poorly, moderately, or highly engaged. A summary of all assessments and the stage at which each will be measured is outlined in Table 1.

### Sample Size

Recruiting 640 participants (16 clusters per arm, 32 clusters in total) will provide 80% power at a 5% level of significance (two-sided) to detect a between-group difference of 15% in the primary outcome at 12 weeks postrandomization, assuming the proportion of participants adherent to healthy lifestyle behaviors in the control group is 30% [43] and an intracluster correlation coefficient of 0.05 [46]. The inflation factor is approximately 3, that is, (1+40−1×0.05), which inflates n=160 per arm in a standard randomized controlled trial to n=480 per arm in a cluster-randomized trial under the same assumption. However, we aim to recruit 1280 participants in total from 64 clusters (32 Māori clusters and 32 Pasifika clusters). This sample size will provide 80% power for the analysis of Māori and Pasifika participants separately if our recruitment target is met.

### Statistical Analyses

Baseline data collected from all participants will be summarized by treatment group, overall, and by ethnic-specific clusters (Māori and Pasifika). Information collected at the cluster level will also be reported. Continuous variables will be presented as numbers observed, means, and SDs. Categorical variables will be presented as frequencies and percentages. Since any differences between randomized groups at baseline could only have occurred by chance, no formal significance testing of baseline differences will be conducted.

The effect of the intervention will be evaluated using an intention-to-treat analysis including all clusters and participants in the group they are randomized to, regardless of whether they receive or complete that treatment. The proportion of participants who are adherent to lifestyle change (≥3 of 4 behaviors) at the end of the 12-week intervention period will be compared between the two treatment groups using generalized linear mixed models with a random cluster effect and adjusting for important baseline confounders. Missing participant data will be taken into account in the mixed model estimates by maximum likelihood, assuming they are missing at random. Similar regression analyses will be conducted on secondary outcomes using the link function appropriate to a continuous or categorical variable. Intracluster correlation coefficients will be estimated. Subgroup analysis will be conducted for Māori and Pasifika clusters separately. Statistical analysis will be performed using SAS 9.4 (SAS Institute Inc, Cary, NC, USA). All statistical tests will be two-sided at a 5% significance level.

**Table 1:** Schedule of enrollment, interventions, and assessments.

Time point		Enrollment (Week 0)		Allocation (Week 0)	Postrandomization (Week 4)	Close-out (Week 12)
**Enrollment**						
	Eligibility screen	X^a^				
	Informed e-consent^b^	X				
	Contact details	X				
**Interventions^c^**						
	OL@-OR@ mHealth tool		X		X	X
	Wait-list control		X		X	X
**Assessments**						
	Age, sex, ethnicity		X		X	X
	Socioeconomic details		X		X	X
	Contact details		X		X	X
	Smoking behavior		X		X	X
	Physical activity		X		X	X
	Alcohol intake		X		X	X
	Fruit and vegetable intake		X		X	X
	Anthropometry (self-reported weight, height)		X			X
	Health status		X			X
	Medication use		X			X
	Kava consumption		X			X
	Holistic wellbeing		X			X
	Pacific and New Zealand heritage and lifestyle^d^		X			X
	Click depth index					X
	Loyalty index					X
	Interaction index					X
	Recency index					X
	Feedback index					X

^a^X indicates the presence of assessments required at particular time periods during the trial.

^b^Consent using a computer-based consent form.

^c^Intervetion was continuous from week 0 to week 12.

^d^Pasifika participants only.

### Engagement Index

Basic descriptive data analysis will be performed on the metrics and components of the engagement index as well as the final index score [44]. To analyze the index score, cutoff points will be developed based on the distribution of the index scores of the total sample using tertiles. Participants will be categorized as either poorly, moderately, or highly engaged. Group comparisons between poorly, moderately, or highly engaged participants will be conducted using generalized linear mixed models. Additional analyses will be performed to determine the association between the index and sociodemographic characteristics of the participants including their education level, ethnicity, age, annual household income, device type (Android or iOS), and system type (app only, Web only, or app and Web).

### Data Management

Data from the trial will be entered into the Drupal database at the study center (National Institute for Health Innovation, University of Auckland, New Zealand). Information about study subjects will remain confidential in keeping with the obligations set out in the Privacy Act 1993, the Health Information Privacy Code 1994 and Section 22B-221 of the Health Act 1956. Access to all study data will be restricted to research staff directly involved in conducting or monitoring the study. Confidentiality will be protected using study registration numbers, and only aggregated and deidentified data will be reported. Computerized information will be password protected, and hard copy information (such as cluster agreements) will be kept in a locked filing cabinet under the responsibility of community coordinators. All reports from the study will be written such that no individuals can be identified. Paper records, electronic files, and source documents will be retained for 6 years from the termination date of the study, in accordance with the requirements of the Privacy Legislation and the Health (Retention of Health Information) Regulations 1996.

### Ownership of Data

Individual study data will remain the property of individual study participants. The study center will have the responsibility for storage, protection, and retrieval of the study data. The University team will have the responsibility for the safe guardianship and use of the data in consultation with the wider project team. The Māori community partners will have guardianship over the data for Māori participants. Access to the data, all analyses, and dissemination of the data will be the joint responsibility of the University team and the Māori Community partners. Māori community partners will review and interpret all analyses involving Māori data and agree on key findings before dissemination.. The same principles apply to data for Pacific participants where Pacific community partners will have guardianship.

### Ethical Approval and Informed Consent

Ethics approval for the trial was obtained from the Northern B Health and Disability Ethics Committee of New Zealand (17/NTB/152/AM01, approved on November 17, 2017). Ethics approval for any amendments to the study protocol will be sought prior to the implementation of the changes (information on the trial registry will be updated accordingly). Maintenance of confidentiality and compliance with the Privacy Act will be emphasized to all study participants. Study participation will be entirely voluntary, and participants may withdraw from the study at any time, without having to provide a reason, by contacting the research team. A participant information sheet and consent form will be given to participants who are identified as being part of a cluster by the local community coordinator during an information meeting. E-consent will be obtained at the time of registration once participants have had the opportunity to read the participant information sheet and ask any questions to their local community coordinator or other members of the study team. If any participants suffer harm from trial participation (which is unlikely), they should be eligible for compensation via their private health or life insurance or via New Zealand’s Accident Compensation Corporation scheme.

### Trial Governance

Trial governance includes a steering committee (on which all authors sit) and a trial management team who will manage the day-to-day processes of the trial, including data management. This trial does not meet two or more of the criteria required for the establishment of a Data Safety and Monitoring Committee [47].

### Dissemination Policy

Results will be disseminated regardless of the magnitude or direction of treatment effect. Dissemination will include adding trial results to trial registration within 1 year of trial completion, feedback to trial participants, publication in an international journal and national and international media releases (including Māori, Pacific, and mainstream media channels) at the time of journal publication, and presentations to participating communities and relevant local, national, and international audiences (including health service funders and providers). In New Zealand, this will include but will not be limited to the Ministry of Health, District Health Boards, Māori and Pacific health provider organizations, general primary health organizations, nongovernment organizations, and health professionals. Criteria for authorship of any papers arising from the trial will be taken from the International Committee of Medical Journal Editors [48].

### Extended Follow-Up of Study Participants

Longer-term follow-up (beyond the 12-week follow-up period of the trial) will be via the New Zealand Integrated Data Infrastructure (IDI) [49]. Participants will be asked if they consent to data gathered in this trial being linked to IDI using their unique national identifier to facilitate longer-term follow-up and assessment of health information that may be influenced by this study, for example, blood tests (blood glucose and cholesterol), visits to health professionals, and diabetes and heart disease medication use. IDI is a large national database containing microdata about people and households in New Zealand. Data is derived from a range of government agencies, Statistics New Zealand surveys, and nongovernment organizations. We have ethics approval to examine such data once a year for a maximum of 5 years after the study ends. All information will be anonymized and will not be linked to information that could identify participants. Written consent will be sought from study participants.

## Results

Trial recruitment opened in January 2018 and will close in July 2018. Trial findings are expected to be available early in 2019.

## Discussion

Most mHealth interventions are designed with minimal input from end-users and lack tailoring to specific (cultural) needs [50]. A culturally-tailored mHealth tool (mobile phone app plus website) aimed at supporting healthier lifestyles among Māori and Pasifika communities in New Zealand was co-designed by an academic and community partnership team [24]. This ground-up work informed a theory-driven approach to content development, including identification of key themes and content domains, selection of behavioral determinants and change techniques, and development of features and functionalities of the mHealth tool.

Comparable user-centered principles were applied to co-designing a community-led, cluster-randomized, wait-list controlled trial to evaluate the impact of the mHealth tool on health behavior change. As such, this project is engaging with communities in a meaningful way throughout the research process, from development to evaluation of the mHealth intervention. This approach has not only resulted in an mHealth tool that aligns with the needs, wants, and lived (cultural) contexts of Māori and Pasifika communities but also anticipated increased engagement and empower communities to make positive lifestyle changes.

## Appendix

Multimedia Appendix 1 SPIRIT checklist.

Multimedia Appendix 2 Trial cluster locations in New Zealand.

Multimedia Appendix 3 Screenshots of OL@-OR@ app.

Multimedia Appendix 4 Motivational Messages.

Multimedia Appendix 5 Control App.

Multimedia Appendix 6 Peer-review report from the funding agency.

## Abbreviations

IDI: Integrated Data Infrastructure

## References

[1] (2014). OECD.

[2] Ministry of Health (2016). Health Loss in New Zealand 1990 - 2013. A report from the New Zealand Burden of Diseases, Injuries and Risk Factors Study. Health Loss in New Zealand 1990 - 2013. A report from the New Zealand Burden of Diseases, Injuries and Risk Factors Study.

[3] Ministry of Health (2013). New Zealand Health Survey Annual Update of Key Findings 2012/13. Annual Update of Key Results.

[4] Theodore R, McLean R, TeMorenga L (2015). Challenges to addressing obesity for Māori in Aotearoa/New Zealand. Aust N Z J Public Health.

[5] Robson B, Harris R (2007). Hauora: Māori Standards of Health IV: A study of the years 2000-2005. Hauora: Màori standards of health IV: A study of the years 2000-2005.

[6] Rush Elaine, Reed Peter, McLennan Stephanie, Coppinger Tara, Simmons David, Graham David (2012). A school-based obesity control programme: Project Energize. Two-year outcomes. Br J Nutr.

[7] Ellison-Loschmann L, Pearce N (2006). Improving access to health care among New Zealand's Maori population. Am J Public Health.

[8] Osei-Assibey G, Kyrou I, Adi Y, Kumar S, Matyka K (2010). Dietary and lifestyle interventions for weight management in adults from minority ethnic/non-White groups: a systematic review. Obes Rev.

[9] Ockene I, Tellez TMR, Rosal Milagros C, Reed George W, Mordes John, Merriam Philip A, Olendzki Barbara C, Handelman Garry, Nicolosi Robert, Ma Yunsheng (2012). Outcomes of a Latino community-based intervention for the prevention of diabetes: the Lawrence Latino Diabetes Prevention Project. Am J Public Health.

[10] Siddiqui F, Kurbasic A, Lindblad U, Nilsson P, Bennet L (2017). Effects of a culturally adapted lifestyle intervention on cardio-metabolic outcomes: a randomized controlled trial in Iraqi immigrants to Sweden at high risk for Type 2 diabetes. Metabolism.

[11] Vincent D, McEwen MM, Hepworth JT, Stump CS (2014). The effects of a community-based, culturally tailored diabetes prevention intervention for high-risk adults of Mexican descent. Diabetes Educ.

[12] Wallia S, Bhopal RS, Douglas A, Bhopal R, Sharma A, Hutchison A, Murray G, Gill J, Sattar N, Lawton J, Tuomilehto J, Mcknight J, Forbes J, Lean M, Sheikh A (2014). Culturally adapting the prevention of diabetes and obesity in South Asians (PODOSA) trial. Health Promot Int.

[13] Gibson A, Miller M, Smith P, Bell A, Crothers C (2013). Institute of Culture, Discourse and Communication.

[14] Statistics New Zealand.

[15] Gorton D, Dixon R, Maddison R, Ni MC, Jull A (2011). Consumers views on the potential use of mobile phones for the delivery of weight-loss interventions. J Human Nutr Dietetics 2011 Online Firstpub Date|.

[16] Hutchesson MJ, Rollo ME, Krukowski R, Ells L, Harvey J, Morgan PJ, Callister R, Plotnikoff R, Collins CE (2015). eHealth interventions for the prevention and treatment of overweight and obesity in adults: a systematic review with meta-analysis. Obes Rev.

[17] Jones KR, Lekhak N, Kaewluang N (2014). Using mobile phones and short message service to deliver self-management interventions for chronic conditions: a meta-review. Worldviews Evid Based Nurs.

[18] Liu F, Kong X, Cao J, Chen S, Li C, Huang J, Gu D, Kelly TN (2015). Mobile phone intervention and weight loss among overweight and obese adults: a meta-analysis of randomized controlled trials. Am J Epidemiol.

[19] Whittaker R, McRobbie Hayden, Bullen C, Rodgers Anthony, Gu Yulong (2016). Mobile phone-based interventions for smoking cessation. Cochrane Database Syst Rev.

[20] World Health Organisation (2011). mHealth: New horizons for health through mobile technologies. Global Obervatory for eHealth series. . Geneva, Switzerland: World Health Organisation.

[21] Flores MG, Granado-Font E, Ferré-Grau C, Montaña-Carreras X (2015). Mobile Phone Apps to Promote Weight Loss and Increase Physical Activity: A Systematic Review and Meta-Analysis. J Med Internet Res.

[22] Ghorai K, Akter S, Khatun F, Ray P (2014). mHealth for Smoking Cessation Programs: A Systematic Review. J Pers Med.

[23] Hall AK, Cole-Lewis H, Bernhardt JM (2015). Mobile text messaging for health: a systematic review of reviews. Annu Rev Public Health.

[24] Khokhar B, Jones J, Ronksley PE, Armstrong MJ, Caird J, Rabi D (2014). Effectiveness of mobile electronic devices in weight loss among overweight and obese populations: a systematic review and meta-analysis. BMC Obes.

[25] Lyzwinski LN (2014). A systematic review and meta-analysis of mobile devices and weight loss with an intervention content analysis. J Pers Med.

[26] Scott-Sheldon LAJ, Lantini R, Jennings EG, Thind H, Rosen RK, Salmoirago-Blotcher E, Bock BC (2016). Text Messaging-Based Interventions for Smoking Cessation: A Systematic Review and Meta-Analysis. JMIR Mhealth Uhealth.

[27] Gandhi S, Chen S, Hong L, Sun K, Gong E, Li C, Yan LL, Schwalm J (2017). Effect of Mobile Health Interventions on the Secondary Prevention of Cardiovascular Disease: Systematic Review and Meta-analysis. Can J Cardiol.

[28] Goodyear-Smith F, Jackson C, Greenhalgh T (2015). Co-design and implementation research: challenges and solutions for ethics committees. BMC Med Ethics Online Firstpub Date|.

[29] Te Morenga L, Pekepo L, Corrigan C, Matoe L, Mules R, Goodwin D, Dymus J, Tunks M, Grey J, Humphrey G, Jull A, Whittaker R, Verbiest M, Firestone R, Ni Mhurchu C (2018). Co-designing an mHealth tool in the New Zealand Māori community with a “Kaupapa Māori” approach. AlterNative: An International Journal of Indigenous Peoples.

[30] Durie M (1994). Whaiora: Maori Health Development. Oxford: Oxford University Press.

[31] Durie M (1999). Te Pae Mahutonga: a model for Maori health promotion. Health Promotion Forum of New Zealand Newsletter.

[32] Durie M, Kingi T (1999). Hua Oranga: A Maori measure of mental health outcomes. Department of Maori Studies, Massey University, Palmerston North.

[33] Pulotu-Endemann FK (2001). Hauora.

[34] Cane J, O'Connor D, Michie S (2012). Validation of the theoretical domains framework for use in behaviour change and implementation research. Implement Sci.

[35] Michie S, Ashford S, Sniehotta FF, Dombrowski SU, Bishop A, French DP (2011). A refined taxonomy of behaviour change techniques to help people change their physical activity and healthy eating behaviours: the CALO-RE taxonomy. Psychol Health.

[36] Michie S, Richardson Michelle, Johnston Marie, Abraham Charles, Francis Jill, Hardeman Wendy, Eccles Martin P, Cane James, Wood Caroline E (2013). The behavior change technique taxonomy (v1) of 93 hierarchically clustered techniques: building an international consensus for the reporting of behavior change interventions. Ann Behav Med.

[37] Godin G, Shephard RJ (1985). A simple method to assess exercise behavior in the community. Can J Appl Sport Sci.

[38] Khaw K, Wareham N, Bingham S, Welch A, Luben R, Day N (2008). Combined impact of health behaviours and mortality in men and women: the EPIC-Norfolk prospective population study. PLoS Med Online Firstpub Date|. pmed.005.

[39] Bush K, Kivlahan DR, McDonell MB, Fihn SD, Bradley KA (1998). The AUDIT alcohol consumption questions (AUDIT-C): an effective brief screening test for problem drinking. Ambulatory Care Quality Improvement Project (ACQUIP). Alcohol Use Disorders Identification Test. Arch Intern Med.

[40] (2018). Ministry of Health.

[41] Kaholokula JK, Nacapoy AH, Grandinetti A, Chang HK (2008). Association between acculturation modes and type 2 diabetes among Native Hawaiians. Diabetes Care.

[42] Tupai-Firestone R, Tuisano H, Manukia M, Kaholokula K, Foliaki S, Kingi T, Kruger R, Breier Bernhard, O'Connell A, Kruger Rozanne, Borman Barry, Ellison-Loschmann Lis (2016). Understanding Pasifika youth and the obesogenic environment, Auckland and Wellington, New Zealand. N Z Med J.

[43] Pfaeffli DL, Whittaker R, Jiang Y, Stewart R, Rolleston A, Maddison R (2015). Text Message and Internet Support for Coronary Heart Disease Self-Management: Results From the Text4Heart Randomized Controlled Trial. J Med Internet Res.

[44] Taki S, Lymer S, Russell CG, Campbell K, Laws R, Ong K, Elliott R, Denney-Wilson E (2017). Assessing User Engagement of an mHealth Intervention: Development and Implementation of the Growing Healthy App Engagement Index. JMIR Mhealth Uhealth.

[45] Peterson E, Carrabis J Vertical Studio. Web Analytics Demystified; 2008.

[46] Adams Geoffrey, Gulliford Martin C, Ukoumunne Obioha C, Eldridge Sandra, Chinn Susan, Campbell Michael J (2004). Patterns of intra-cluster correlation from primary care research to inform study design and analysis. J Clin Epidemiol.

[47] Fleming T, Demets Dl, Fleming Tr, Ellenberg Ss, Ellenberg (2002). Data monitoring committees in clinical trials: a practical perspective. Data Monitoring Committees In.

[48] International Committee of Medical Journal Editors.

[49] (2017). Statistics New Zealand.

[50] Eyles H, Jull A, Dobson R, Firestone R, Whittaker R, Te Morenga L, Goodwin D, Mhurchu Cn (2016). Co-design of mHealth Delivered Interventions: A Systematic Review to Assess Key Methods and Processes. Curr Nutr Rep.

