# Supplementary figures and images for: A Co-Designed, Culturally-Tailored mHealth Tool to Support Healthy Lifestyles in Māori and Pasifika Communities in New Zealand: Protocol for a Cluster Randomized Controlled Trial
Source: JMIR Res Protoc. 2018 Aug 22;7(8):e10789. doi: 10.2196/10789 (PMC6125615; doi:10.2196/10789)

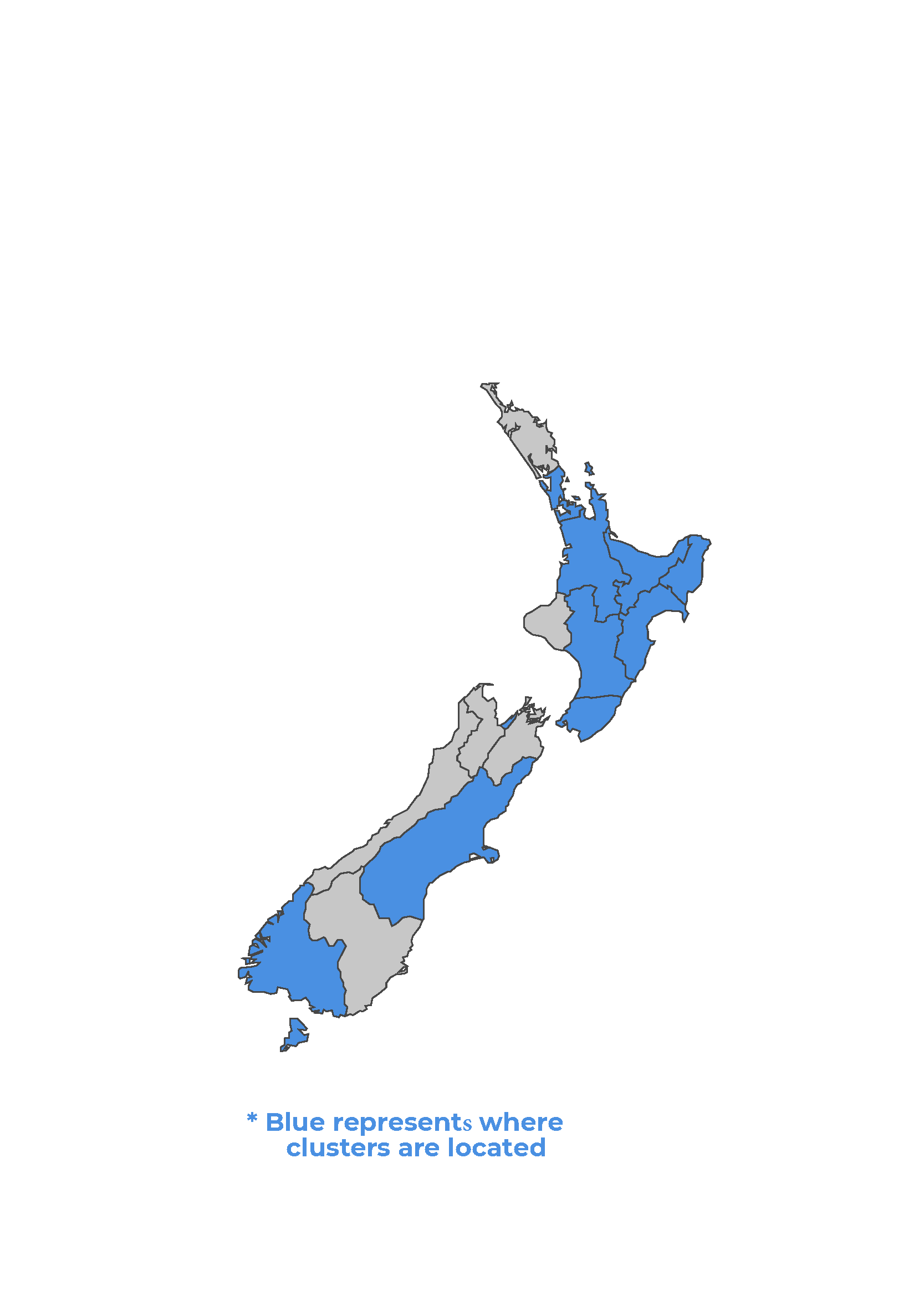

Supplement: Multimedia Appendix 2 [file resprot_v7i8e10789_app2.png]

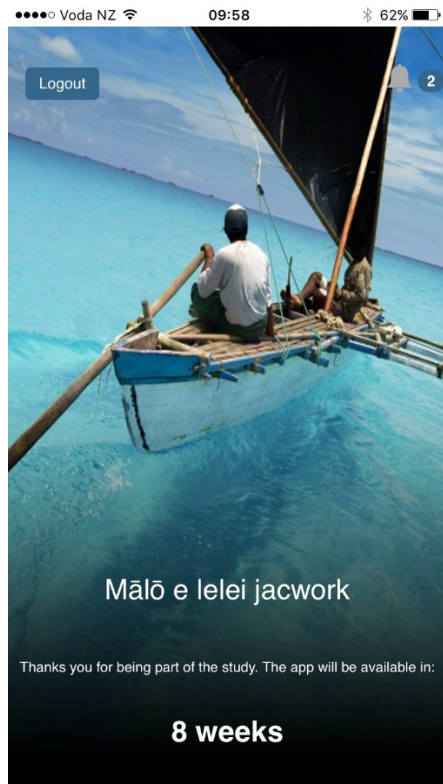

Figure 1: Pasifika version of the control app

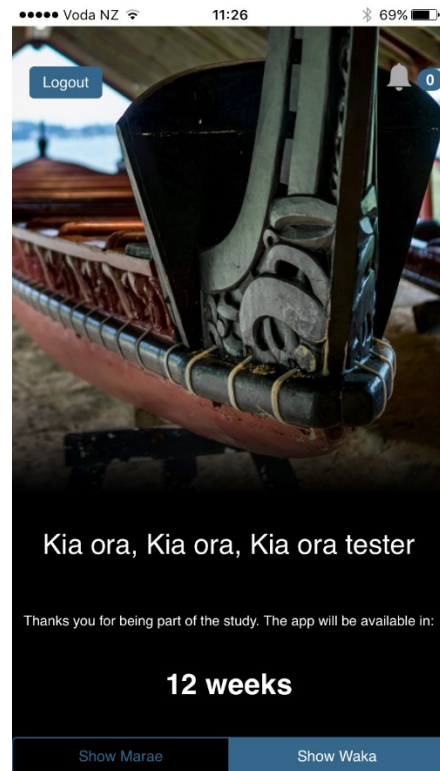

Figure 2: Māori version of the control app

Supplement: Multimedia Appendix 5 [file resprot_v7i8e10789_app5.pdf]
