# Supplementary material for: A Co-Designed, Culturally-Tailored mHealth Tool to Support Healthy Lifestyles in Māori and Pasifika Communities in New Zealand: Protocol for a Cluster Randomized Controlled Trial
Source: JMIR Res Protoc. 2018 Aug 22;7(8):e10789. doi: 10.2196/10789 (PMC6125615; doi:10.2196/10789)

Goal setting

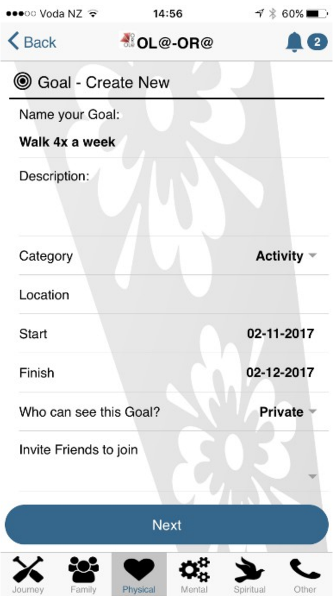

1.

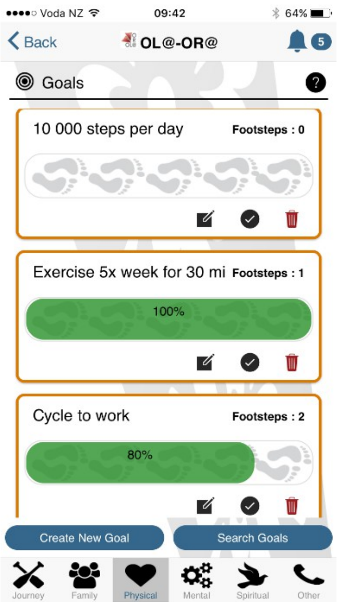

2.

Key:

1. Creating a goal (wero)
2. Reviewing goals and updating progress
3. Rewards for achieving goals (Māori version)
4. Achieving 5 goals to receiving a taonga (treasure such as a tiki shown in picture) (Māori version)
5. Rewards for achieving goals (Pasifika version)
6. Achieving 5 goals to receive a fully grown coconut tree (Pasifika version)
7. Physical page - Physical activity and food sections
8. Food of the Atua – cultural importance around food and helpful tips with building a vegetable garden, eating seasonal foods and looking for local markets (Māori version)
9. Search physical activity options by region
10. Food tracker
11. Step tracker
12. Help and support – links to other services

Rewards for achieving goals

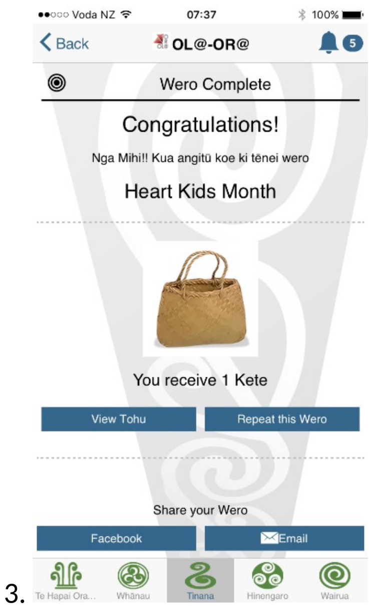

3.

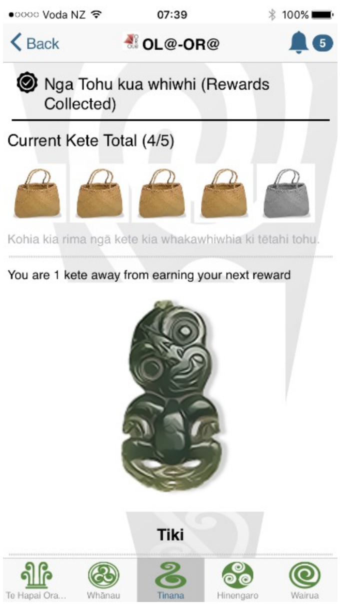

4.

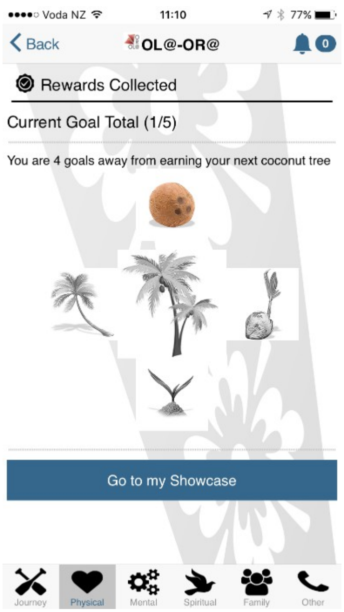

5.

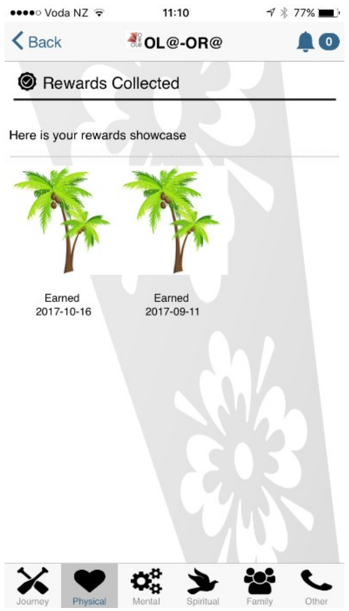

6.

Physical activity and healthy eating section

7.

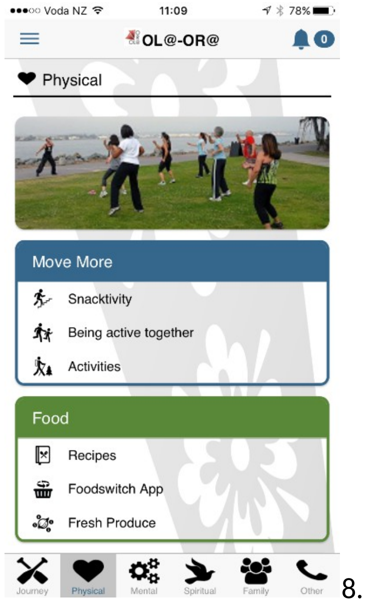

8.

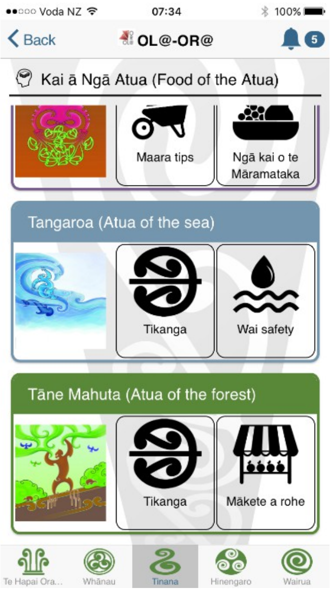

9.

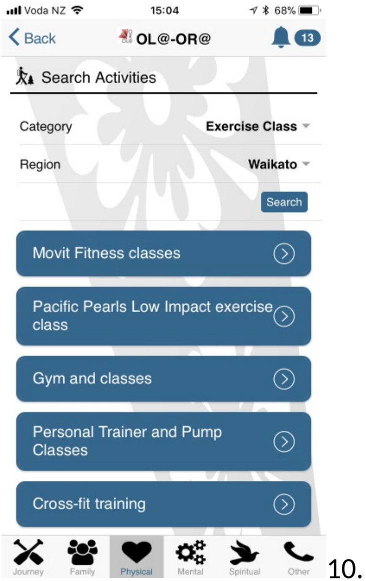

10.

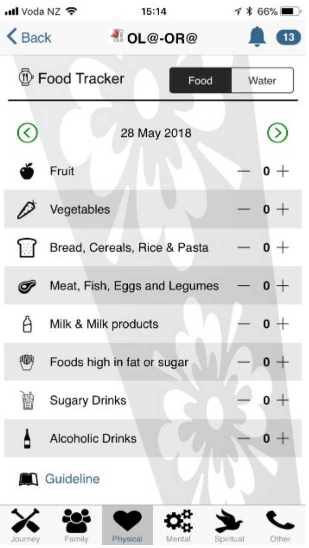

11.

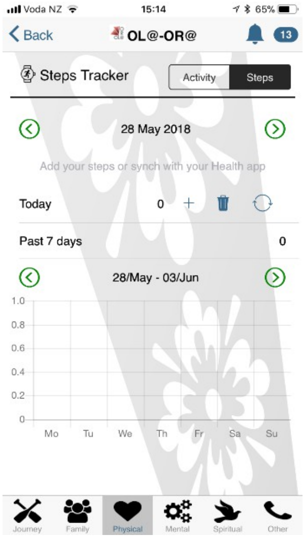

Contact details for other services

12.

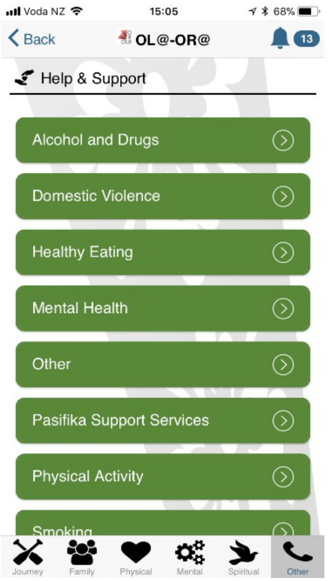

Supplement: Multimedia Appendix 3 [file resprot_v7i8e10789_app3.pdf]
