# Supplementary material for: A Co-Designed, Culturally-Tailored mHealth Tool to Support Healthy Lifestyles in Māori and Pasifika Communities in New Zealand: Protocol for a Cluster Randomized Controlled Trial
Source: JMIR Res Protoc. 2018 Aug 22;7(8):e10789. doi: 10.2196/10789 (PMC6125615; doi:10.2196/10789)
Supplement: Multimedia Appendix 4 [file resprot_v7i8e10789_app4.pdf]

Type of message: Motivational/ Whakatauki

Received on: Mondays

Example 1 (Māori): *Whāia te iti kahurangi ki te tūohu koe me he maunga teitei (Seek the treasure you value most dearly: if you bow your head, let it be to a lofty mountain - be persistent, don't let obstacles stop you from achieving your goals)*

Example 2 (Pasifika): *You don't have to see the whole staircase, just take the first step*

Type of message: Lifestyle Message

Received on: Wednesdays

Example 1 (Māori): *Feeling stressed? Going for a hikoi, eating healthy and spending time with friends and whānau are all good ways to help relieve stress.*

Example 2 (Pasifika): *Feeling stressed? Going for a walk, eating healthy and spending time with friends and family are all good ways to help relieve stress.*

Type of message: Smoking Cessation Message

Received on: Thursdays (if a smoker)

Example 1 (Māori): *Quitline offers free advice and support to quit smoking. Call 0800 778 778 or go to their website*

Example 2 (Pasifika): *Quitline offers free advice and support to quit smoking in Samoan, Cook Island Māori, Tuvaluan, Tongan and Tokelauan languages. Just call them on 0800 778 778*

Type of message: Spotlight on Function Messages

Received on: Fridays

Example 1 (Māori): *Don't forget to invite your friends and whānau to join you on your journey to better health by visiting the Whānau section of the app.*

Example 2 (Pasifika): *Don't forget to invite your family/friends to join you on your journey to better health by visiting the Family section of the app.*

Type of message: Goal review messages
Received on: Sundays

Example 1 (Māori): *Kei te pai, you have been using OL@-OR@ for a week! Don't forget to review your wero & ensure you are making positive steps towards a healthy lifestyle.*

Example 2 (Pasifika): *Well done, you have been using OL@-OR@ for a week! Don't forget to review your goals & ensure you are making positive steps towards a healthy lifestyle*
