# Supplementary material for: A Co-Designed, Culturally-Tailored mHealth Tool to Support Healthy Lifestyles in Māori and Pasifika Communities in New Zealand: Protocol for a Cluster Randomized Controlled Trial
Source: JMIR Res Protoc. 2018 Aug 22;7(8):e10789. doi: 10.2196/10789 (PMC6125615; doi:10.2196/10789)
Supplement: Multimedia Appendix 6 [file resprot_v7i8e10789_app6.pdf]

Healthier Lives – He Oranga Hauora National Science Challenge

FEEDBACK TO PROJECT TEAMS ABOUT RESEARCH PROPOSALS

Your project has been selected for inclusion in the 1st tranche of contracted research in the Healthier Lives Science Plan. All projects to be included in the Science Plan are now asked to:

1. **Revise your budget:**
   a) Projects will be funded for 3 years only. Please delete Years 4 and 5 from the budget but don't increase the total funding for Years 1-3! (You will have the option to spend your funding over a longer period than 3 years but need to present a budget for 3 years only.)
   b) Projects must have a PI at 0.2 FTE (minimum), who will be responsible for leading the project, and accountable for reporting on expenditure and achievement of milestones.
   c) Projects must have no more than 0.3 FTE for other investigators. If currently above 0.5 total FTE for PI/AIs, please reduce the funded FTEs in your project to this level. (If under 0.5, please do not increase your FTEs to this level unless you can make the equivalent savings elsewhere in your budget – the total requested for Years 1-3 must not increase).

2. **Update the 'Project Team Leaders/Collaborators' table:**
   a) The 'Project Team Leaders/Collaborators' table needs to show FTEs for all staff involved with the project, including those who are not PI/AI/Advisors, eg Project Manager, Community Researcher, App Developer etc. This table should indicate if any FTEs are funded from other sources.
   b) All staffing FTEs listed in this table should be clearly identified in the budget.

3. **Add milestones:**
   a) Please add a section entitled 'Milestones' to your proposal, which specifies milestones that will be achieved at: 6 months; 18 months; and 24 months. These should be realistic deliverable outputs that can be reported to MBIE. **Please note that release of funding at 6 monthly intervals may be contingent on achieving these milestones.**

4. **Complete the tables in the attached document and send to Jean by no later than MONDAY 11 MAY 2015.**

5. **Revise and re-submit your proposal by no later than MONDAY 11 MAY 2015:**
   a) Please specifically address the feedback from the NZ Advisory Panel (highlighted in bold below) when revising your project.
   b) Please review the feedback on your proposal from the Science Advisory Panel and Stakeholders (see below), and revise your proposal where necessary/possible.

Feedback from NZ Advisory Panel:

- **Several stakeholders/advisers have identified the importance of your project's inter-Challenge links with the He Pikinga Waiora project. You will need to give careful thought to a realistic alignment of the timelines of both projects so that you can achieve your stated intention of 'apply[ing] their implementation framework for activating communities to our engagement with Maori and Pacific communities'.**

- **More details about the process of app development would be useful. Who will undertake this work and what is their track record in this area?**

- **You have a specific timeframe for your project, with different stages happening in different years, but this is not reflected by any significant variation in the budget from year to year. It would be helpful to understand the nature of the various subcontracts in the budget, especially the largest one, and this might be easier if they were not described as 'subcontracts' but in more specific terms.**

<u>Feedback from Science Advisory Panel and Māori Advisor:</u>

- Restricted but clear and important goal. The instrument can be of good use for the Healthier Lives purpose - also for Maoris.

- This proposal is excellent on all three criteria. mHealth itself has a high potential reach and novelty. The study focuses directly on Maori and Pasifika populations and involves participants in co-designing the intervention (s), and the proposed approach has been piloted and found promising. Another key strength is the focus on individuals. Although environmental and policy changes are extremely important, the ultimate pathway for these interventions to work involves individual behaviour change. Widespread dissemination of counselling that can increase uptake of available opportunities for healthy living is therefore needed. Synergies with other types of interventions seem probable. Additional positives are the adaptive design that allows re-randomization of non-responders as well as the capture of 'Big Data'. The adaptive design has the inherent advantage of the potential to test several different interventions that might appeal differentially to members of populations of interest. Regarding the design, I was curious about the fixed time element of 6 or 12 months for the trial of an intervention and also about the potential for those initially successful to revisit the same or a different intervention after the initial period. Fixed intervention durations are common in other behavioural intervention formats but it was less clear to me that this is essential or optimal for mHealth trials. The rationale for 6 or 12 months was given, i.e., it is long enough to see if there is going to be a response, but the proposal was less clear on whether or how ongoing support for behaviour change and maintenance would be provided. Regarding capturing passive data on weight and blood pressure I was curious as to the level of development of this technology, particularly with respect to blood pressure and wondered whether the data quality could be a concern. We were asked to comment on the question of whether this project should refocus on Maori and Pasifika populations at high risk of obesity. Given that the proposal already focuses on Maori and Pasifika populations, I take this question to contrast focusing on these populations in general vs. only those at high risk. I suppose to do that would alter the recruitment strategy to focus on populations with identified risk factors. Not sure how this would complicate things. Overall, I think that an overall population focus is appropriate although planned subgroup analyses of those who turn out to be at high risk could enhance the design, particularly if risk status turns out to relate to the need for re-randomization.

- As mentioned by the applicants engagement with He Pikinga Waiora is essential for any successful outcome

- The proposal has potential to reduce obesity through a novel means of communication utilizing mobile and wireless technologies as a potential solution to the problem of behavioral change with respect to projected increasing rates of obesity. Whereas the intervention is cost effective and is projected to have positive effects based on evidence from other previous work, it does seem narrow in application with a focus on obesity alone and could be more broadly targeted to a range of connected risk factors predisposing towards chronic conditions and illness prevention. It may also be helpful to provide the mobile phone technology to those who cannot afford the devices recognizing the opportunity to intervene in the low social economic strata that would likely be attracted by the

use of a mobile phone and be able to receive the health promotion messages that may otherwise not be communicated. Perhaps the mobile and wireless industries could partner with the project to increase their image as good corporate citizens.

- The proposal has potential and is cost effective it has also proven to have a positive effect through previous research but limited in focus and maybe not hitting the high risk hard to engage communitities that cant afford the tecnology etc. Engagement with the He pikinga Waiora is essential to their success.

- The proposal has potential and is cost effective it has also proven to have a positive effect through previous research but limited in focus and maybe not hitting the high risk hard to engage communitities that cant afford the tecnology etc. Engagement with the He pikinga Waiora is essential to their success.

Feedback from Stakeholders:

Q *The proposed research has been designed for participants from a Māori and/or Pasifika background with a BMI of at least 30 but should we re-focus it on those more at risk?*
A No, based on experience, it's better to focus research on participants who want to be involved. Those at higher risk often have such chaotic lives they may not be able to participate.

Q What is the cost of this type of intervention? Is it cheaper than medical treatment?
A *Determining the cost-effectiveness is part of the research. Text messages cost .20c or less. The model proposed is similar to that of the Smoking Quitline. It's envisaged as highly accessible, easily scalable to a larger population, and hopefully very cost-effective.*

Q Will there be real people actually sending out/answering texts?
A *No, that would be expensive. But we know people do want a highly personalised response that's individual to them. Algorithms will be used to produce text messages that are tailored to their age, gender and interests, and there is also the opportunity to have blogs, social media and games for engagement with other people.*

Q What will you do if participants in this co-design research ask for something that is a) not cost effective or b) outside your control, eg environmental change?
A *It's possible they might say that what we can provide is not what they want, and we would learn from that.*

Q MHealth hasn't always gelled with Māori communities in the past, possibly because it is too generic and dry, so it's really pleasing to see the co-design approach, which I strongly support. The initial focus on Māori and Pasifika is good – as I travel up and down the country I see that people are screaming out for this kind of weight management intervention and there are lots of synergies with other projects. Relationships will be a crucial to enable co-design to work well – how will you engage and can we help with that?
A *This is the first of many conversations — we'll need to work with you and other Māori and Pasifika providers.*

C It will be better to frame this type of intervention as 'support from someone walking alongside me' rather than 'advice from on high'.
R *That's right. In previous work on quitting smoking we buddied up participants. It will be exciting to develop these ideas further.*

Q Could the focus on Māori and Pasifika be broadened to include refugees and other migrant populations?

A *We are starting with Māori and Pasifika because that's the feedback we received at previous consultations. We know that mobile phone ownership doesn't vary markedly by socio-economic group. Health literacy will also be an important factor. The program will eventually be rolled out for other ethnic groups.*

W This is a good, innovative, timely proposal which addresses a need in the wider community/those affected disproportionately by NCDs. I note it is for those 18 years and over, but there have also been major increases in obesity in adolescents, who are also users of the technology and although often considered hard to reach in a 2008/09 survey of 5-24 year olds a third of them wanted to lose weight and further analyses of the data also showed that adolescents who were overweight/obese also wanted to lose weight. I also note the interventions are for 6 or 12 weeks and we know that most people can lose weight especially if they are part of a programme being followed-up but most challenging is ensuring people do not regain the weight lost. So I think this would need to address how people are assisted after the intervention or how they can re-enter a programme easily if they have regained the weight lost that focuses on maintaining the weight loss, even if modest, in the longer term.

W The action, reflection and adaption process necessary for effective implementation into practice was particularly evident in the personalised prevention using new technologies and the He Pikinga Waiora – making health interventions work for Māori communities' proposals. This area is one that the HRC does not normally support – and there is a great need for this type of understanding with an ongoing approach able to be adapted to suit "right treatment and treatment" to the "right population" in the context of their everyday lives. i.e. Implementation science

W There was no discussion with the HPA research team prior to presentation of this proposal. A four year timeframe carries project risks that have not been accounted for. Consider what the uptake of smartphones was in 2011. What sort of burden is being applied by 'app' fatigue? Further project risk include low uptake by marginalised sector groups there by over representing population health impacts. It is not clear what 'big data' is being referred to – nor what biosensors are relevant. I am not convinced the proposal needs to be funded for 4 years. The main independent variable is 6-/12 weeks – however not sure whether such a project is culturally appropriate or well tailored – this is weak. It is a well constructed project but does seem to be repackaging a concept with embedded assumptions about the success of the method—what is the real research question? It is really nice to see the analysis frame and the power analysis. Two things being tested at once: culturally tailored Apps and 6/12 weeks of app trial. The proposal does not distinguish how one might confound the other leading to a very poor representation of the proposed research method.

Key: Q = question; A = answer; C = comment; R = response; W = written comment
